# Nutritional Value of Meals Designed for a School-Based Food Aid Program and Comparison with Similar Commercial Products: An Example of Good Practice from the DIATROFI Program

**DOI:** 10.3390/children10071268

**Published:** 2023-07-23

**Authors:** Matina Kouvari, Dimitrios V. Diamantis, Konstantinos Katsas, Vasiliki Radaios, Afroditi Veloudaki, Athena Linos

**Affiliations:** 1 PROLEPSIS Civil Law Non-Profit Organization of Preventive Environmental and Occupational Medicine, 15121 Athens, Greece; m.kouvari@prolepsis.gr (M.K.); d.diamantis@prolepsis.gr (D.V.D.); k.katsas@prolepsis.gr (K.K.); v.radaios@prolepsis.gr (V.R.); a.veloudaki@prolepsis.gr (A.V.); 2 Department of Nutrition and Dietetics, School of Health Science and Education, Harokopio University, 17676 Athen, Greece; 3 Discipline of Nutrition and Dietetics, Faculty of Health, University of Canberra, Canberra, ACT 2601, Australia; 4 Functional Foods and Nutrition Research (FFNR) Laboratory, University of Canberra, Bruce, ACT 2617, Australia; 5 Medical School, National and Kapodistrian University of Athens, 11527 Athens, Greece

**Keywords:** DIATROFI Program, school meals, food assistance program, children

## Abstract

Providing meals of high nutritional value should be the principal objective of large-scale school-based food aid programs. This study aimed at highlighting the nutritional value of meals distributed in the school-based food assistance DIATROFI Program by comparing them to their commercially available counterparts. For the purpose of this study, *n* = 13 DIATROFI meals and *n* = 50 commercial products from the 2016–2017 school year, and *n* = 12 DIATROFI meals and *n* = 40 commercial products from the 2022–2023 school year were selected. The protein, carbohydrate, total sugar, dietary fiber, total fat, sodium/salt content, and fatty acid methyl ester profile of DIATROFI meals were estimated through recipe simulation and national/international food databases, and verified through laboratory analyses while the relevant information was extracted from the label for commercial products. As verified by laboratory analyses and in comparison with food labels, most DIATROFI meals had lower total fat, saturated fatty acid, and sugar content, and most had higher dietary fiber content during both years. Many recipes’ nutrient profiles also improved over time. DIATROFI meals present significant advantages over available commercial products. Such tailored-made school meals can prove to be advantageous in terms of nutrition profile compared to commercially available, which have yet to be impacted by food reformulation.

## 1. Introduction

Considering that the burden of non-communicable diseases (NCDs) is rapidly increasing worldwide, there is an imperative need to take action to encourage lifestyle modifications, as demonstrated in the WHO Package of Essential Non-Communicable Disease Interventions For Primary Healthcare [1]. A diversified, balanced, and healthy diet early in the life course helps prevent all forms of malnutrition, as well as a wide range of NCDs [2]. Food aid programs within schools, especially in countries with a protracted financial crisis, are usually adopted as a policy measure to address the aforementioned needs [3].

In this context, the Prolepsis Institute developed a novel school-based food aid program, DIATROFI (http://diatrofi.prolepsis.gr/en/ (accessed on 22 May 2023)), for a dual purpose: firstly, to reduce food insecurity by providing all students of participating schools with free, healthy, Mediterranean diet-based and safe-for-consumption meals daily, and secondly, to promote healthy nutrition educational activities in students and their families [4].

Evidence-based guidelines of food and nutrition practitioners aimed at improving children’s well-being recommend avoiding energy-dense and nutrient-poor food/beverages usually provided in the global market and school canteens [3]. The DIATROFI meals are designed particularly for the Program because comparable commercial products do not fulfill the Program’s strict safety and nutritional requirements. An equally important aspect is the appropriate assessment of the nutritional value of the DIATROFI meals. The most usual nutrient content evaluation is adopted from national/international databases based on the recipe simulation of packed/pre-packed meals [5,6]. Nonetheless, important biases have been documented regarding this method [7]. Therefore, there is an increase in demand for more accurate and efficient methods that accompany EU legislation [8] for a more precise nutritional assessment of food labels. In this context, conducting analyses in laboratories accredited through ISO 17025:2005 is to provide more reliable information on the nutritional composition of DIATROFI meals.

The primary purpose of the present work is to directly compare the nutrient content of meals exclusively designed and distributed in the context of the DIATROFI Program with the nutrient content of similar commercially available packed/pre-packed ready-to-eat meals in the market across two schoolyears six years apart. The secondary purpose is to verify the nutrient values of DIATROFI meals as presented in food suppliers’ nutrition facts through laboratory analyses.

## 2. Materials and Methods

### 2.1. DIATROFI’s Program Design

The design, methodology, and sampling procedure of the DIATROFI Program are presented elsewhere [4,9]. In brief, the DIATROFI Program is a school-based food-assistance Program that aims to tackle the high food insecurity among students in Greece while also improving their dietary habits following the Mediterranean dietary pattern. Children receive a daily meal each school day, and since 2012, approximately 123,000 students have received 18,000,000 meals in more than 880 Greek schools.

### 2.2. DIATROFI School Meals

To design the meals distributed in the DIATROFI Program, international age-specific nutritional guidelines are taken into consideration, including the European Food Safety Authority (EFSA) [10], Dietary Reference Values (DRVs), the United States Department of Agriculture (USDA) Nutrition Standards for School Meals [11], the World Health Organization (WHO) Food and Nutrition Policy for Schools [12], and recent advances in nutritional sciences. In addition, the meals are adapted to participating students’ age-specific nutritional needs and adhere to the principles of the traditional Greek diet, as detailed in the National Dietary Guidelines for infants, children, and adolescents [13].

The daily meal is composed of a sandwich or traditional savory pie, a dairy product, and fresh fruit(s). Meals are designed according to the following requirements: (a) exclusive utilization of extra virgin/virgin olive oil as the only added source of fat, (b) use of >60% whole grain flour, of the total flour used, in all bread/bakery products, (c) avoidance/absence of added preservatives and artificial additives, as well as the addition of only the necessary amount of natural additives for meal production, (d) low sodium and sugar content, and (e) absence of artificial or natural sweeteners.

### 2.3. Collection of DIATROFI Meal Samples’ Food Labels during Two School Years

Although school meal samples are collected regularly on an annual basis, for the purpose of this study, we utilized the data from meal samples collected across two school years, six years apart. More specifically, we compared the meal samples for the school year 2016–2017 and school year 2022–2023. Limited samples from the school year 2021–2022 were also included (two cakes). The food label data for total fat, saturated fat, carbohydrate, sugar, protein, dietary fiber, and sodium were collected for all available products.

### 2.4. Collection of DIATROFI Meal Samples for Laboratory Analysis

Random representative meal samples are collected by Prolepsis Institute members during school visits for various sensory-based and laboratory analysis tests. Sampling is based on appropriate standards (e.g., sealed to prevent potential fraud, having all the appropriate information for sample identification/traceability). All samples are placed into isothermal packs with ice coolers, accompanied by the traceability information, and are immediately transferred to accredited food laboratories without previously notifying the supplier.

### 2.5. Laboratory Analyses: Sample Preparation & Analytical Methods (2016–2017)

Laboratory analyses of (a) ready-to-eat meals received from schools and (b) raw materials and ready-to-eat meals received directly from the food supplier during unannounced inspections were performed. All samples are monitored for safety by examining the existence of harmful chemicals and microorganisms. For the 2016–2017 school year, additional laboratory analyses for seven nutrient components were performed on DIATROFI meals (*n* = 13).

All solvents and reagents used for sample preparation were of analytical grade. After the appropriate preparation, all analytical samples were weighed, appropriately homogenized, and stored at −18 °C until analysis, which was performed between approximately 7 to 10 days of storage.

All results were obtained by applying official analysis methods used by the cooperated Food Laboratories in Greece. More specifically, total protein (after sample homogenization, digestion with acid, and distillation and titration of NH^4+^) is determined through the ISO 1871:2009 (Kjeldahl) method, and total fat (after sample homogenization and insertion in Soxhlet Thimble) is extracted with petroleum ether under heat for 6 h and gravimetrically quantified [14,15]. The fatty acids profile (FAME) (through the use of the fat extracted and methylation of fatty acid) is performed through gas chromatography with a flame ionization detector (GC-FID) [15,16]. Total dietary fibers are analyzed through the AOAC 985.29 enzymatic-gravimetric method (including homogenization, dry and de-fat of the sample, enzymatic reactions through a-amylase/amyloglucosidase/protease, filtration, the weight of the sample, the first sample used for inorganic materials determination and second sample for remaining nitrogen through Kjedahl and calculation) [17]. The sugar content (after sample homogenization, sugars extraction with water and filtration) is quantified through high-performance liquid chromatography (HPLC) and the Refractive Index Detector, and the salt content (after sample homogenization and digestion with acid) is determined through the application of Atomic Absorption Spectrometry (A.A.S), including flame for samples with low salt content or titration (according to Mohr method) for samples with high salt content (>0.5%) [18,19]. The amount of total carbohydrates is obtained by subtracting the total amounts of water content, total protein, total fat, and ash from 100.

In order to obtain repeatability of the analytical result for each nutrient analysis, 3 replicates of independent samples of the same meal were analyzed, resulting in a mean value and its relative standard deviation (RSD). As a result, *n* = 13 × 3 = 39 meal samples were collected, and *n* = 39 × 7 nutrient components = 273 laboratory analyses were performed. The independent results for each meal sample × nutrient were obtained through the same analytical method under the same analysis terms (i.e., same operator, apparatus, and instruments in the same laboratory) [20].

For each sample analyzed, the Prolepsis Institute requested the storage of a counter sample under freezing conditions (−18 °C) for at least a month after the laboratory report, allowing for the potential of a repeated analysis in case of significant deviations of the analysis compared with the results obtained through national/international databases and food labels.

### 2.6. Evaluation of Nutrient Content of DIATROFI Meals through National and International Databases

The nutrient content of the distributed DIATROFI school meals was estimated on the basis of 100 g. For each one of the meals, raw materials and ingredients recorded on the food label, along with their exact proportional content to the ready-to-eat product, are utilized. National and international food databases are used to calculate the macro- and micro-nutrient content of each meal. International food databases, such as the Cyprus Food Composition Tables [21], as well as McCance and Widdowson’s Composition of Foods, and the United States Department of Agriculture nutrient database [22,23] were used, along with data from Greek commercially available foods. Energy, protein, total carbohydrates, total sugars, total fat and fatty acid methyl ester (FAME) profile, dietary fibers, sodium, and vitamins A, D, E, C, folate, and B12 are calculated. Considering the purposes of the present work, only the macronutrient and sodium content of the DIATROFI meals across two school years (2016–2017 and 2022–2023) is presented, as it is demanded in EU food labels through EU 1169/2011 legislation [8].

### 2.7. Collection of Commercial Meal Samples

Sampling was also performed for commercially packed/pre-packed meals. In 2016 and 2023, the Prolepsis Institute’s Dieticians and Food Technologist visited 4 different supermarket chains and 3 bakery restaurant/cafés chains, searching for meals comparable to DIATROFI meals of different brand names in the Greek trade market. A total number of 30 in 2016 and 37 in 2023 ready-to-eat packed or pre-packed meals (3 to 5 per meal) were collected, with all the appropriate food labeling, according to legislation, including information about (a) ingredients/additives and (b) quantities (g or mg/100 g of the final meal) for each nutrient compound. The main selection criteria included sales figures and details on the food label. Furthermore, an extensive search was performed for comparable meals found in EU global markets that are unavailable in the Greek market, resulting in an additional 20 nutrition labels for 2016 and 3 for 2023 (apart from two products where no similar commercially available sample was available at 2016); these are also presented in the four Tables. In total, four Tables were produced, one for traditional pies, one for bakery products, one for whole wheat bread and dough products (i.e., pizza), and one for sandwiches.

## 3. Results

### 3.1. DIATROFI’s Meals’ Nutrient Content

The nutrient content (i.e., total fat, saturated fatty acids, carbohydrates, dietary fibers, total sugars, and sodium) of DIATROFI meals derived from the 2016 laboratory analysis, 2016 and 2023 food labels, and 2016 and 2023 food database analysis, along with comparable commercially available products from 2016 and 2023 are presented in the Tables. Overall, most DIATROFI meals had lower total fat, saturated fatty acid, and total sugar content and higher dietary fiber content compared to commercial products. Simultaneously, most of the similar commercially available and highly available-in-market products have made little to no improvement in their nutrient content.

### 3.2. Comparison between DIATROFI Meals and Similar Commercially Available Products (Food Labels and Food Databases)

#### 3.2.1. Traditional Pies, including Vegetables and/or Cheese and/or Chicken

All traditional pies were of lower fat and saturated fat content than commercially available products across both school years (2016–2017 and 2022–2023), apart from the newly introduced chicken pie, due to added extra virgin olive oil (Table 1). Throughout the years, the sugar content was lower in DIATROFI’s leek pie and in the spinach pie (with and without feta cheese) following an update in the ingredient content, compared to commercially available products. The rest of the pies were similar in their sugar content. The protein measured through nutritional labels was slightly lower for most DIATROFI pies, in contrast with the dietary fiber, for which the content in DIATROFI’s pies was higher. The protein and sodium content of DIATROFI’s pies were lower than most commercially available products, mostly due to the high quantities added extra virgin/virgin olive oil, which was not included in most commercially available products and thus did not increase the total product’s weight.

#### 3.2.2. Bakery Products

DIATROFI meals’ total fat, saturated fat, and sugar were lower compared to commercially available products in 2016 and 2023 (Table 2). Sodium content was similar between DIATROFI’s meals and commercially available products; however, following recipe adjustments, the sodium content between DIATROFI’s must cookies was quite lower than commercially available must cookies. Protein content was higher in DIATROFI’s cakes than in commercially available products in 2016 and 2023.

#### 3.2.3. Whole Grain Bread and Dough Products

The whole grain bread provided by the DIATROFI program exhibits a preferable nutrient profile than similar commercially available products in 2016 (old product) and 2023 (new product) in terms of less fat, saturated fat, sugar, and sodium (Table 3). No notable differences were noted in the newly added DIATROFI pizza products and similar commercially available products, except for the lower sodium content of the DIATROFI pizzas and significantly higher fiber in DIATROFI’s whole grain pizza with chicken. It should be noted that most commercial products were frozen, meaning that when heated, they will lose water, and all nutrient content, at 100 g, will increase.

#### 3.2.4. Sandwiches 

All DIATROFI sandwiches reported higher content of dietary fiber and lower sodium content. Protein content was higher for the DIATROFI chicken tomato sandwich and egg with olive oil sandwich (Table 4). All sandwiches were discontinued for the schoolyear 2022–2023 due to limited acceptability and exceptionally high production costs compared to their offered nutrients.

### 3.3. Comparison between DIATROFI Meals and Similar Commercially Available Products (Laboratory Analysis Results 2016–2017)

We evaluated the differences in nutrient content between DIATROFI meals and commercially available product labels, as the laboratory analyses are more credible at identifying the true nutrient content. Regarding the Greek traditional pies, those prepared in the context of DIATROFI vs. commercial pies had notably less total fat and approximately three times less SFAs (i.e., spinach pie 0.9 g in DIATROFI vs. 4 g in similar commercial products) (Table 1). In all bakery products, total sugar content was at least 1.5 times higher in commercially available products compared to those exclusively prepared for DIATROFI (i.e., first cake with vegetables 10.9 g in DIATROFI vs. 35 g in similar commercial) (Table 2 and Table 3). Similar results were observed for sandwiches, as they had almost double the dietary fibers when compared with the respective commercial ready-to-eat meals (Table 4). Regarding sodium, all DIATROFI meals contained lower amounts compared to commercially available products.

### 3.4. Laboratory Analyses, Food Labels, and Food Databases Results

Nutrition labels and food databases tend to overestimate the actual fat content in most meals and underestimate protein and dietary fiber, up to twice as much. For example, spinach pie (with and without cheese) and leek pie had higher total fat and SFA and less protein and dietary fiber based on their nutrition label in comparison with the laboratory analysis (Table 1). When the nutrient content was examined throughout the evaluation of the ingredient’s nutritional profile, food databases tended to overestimate most food labels’ nutrient content. Food databases and laboratory analysis results were almost similar (±0.5 g) across most products and nutrients. 

### 3.5. Fatty Acids Methyl Ester Profile

**Figure 1:**
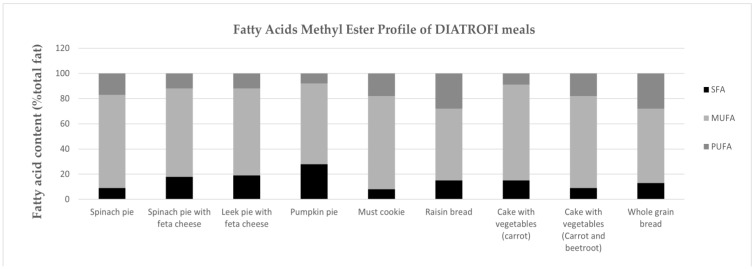
Fatty acids methyl ester profile of school meals exclusively designed for the DIATROFI Program, estimated through laboratory analysis (2016).

In Figure 1, the FAME profile of DIATROFI meals, based on the laboratory report statement, is illustrated. Overall, DIATROFI meals had, on average, 70% MUFA (range 57–76%), 15% PUFA (range 8–28%), and 15% SFA (range 8–28%) of the total fat.

## 4. Discussion

### 4.1. DIATROFI Meals

In the present work, the nutritional value of the DIATROFI meals, verified partially through analytical methods from Accredited Laboratories, was evaluated. Most importantly, the discrepancies between the nutrient content of meals exclusively designed and distributed in a large-scale school-based food aid program compared to commercial products available in the market and usually used in school canteens are highlighted. Overall, DIATROFI’s meals were superior in terms of their nutrient profile and provided healthy meals with limited saturated fats, sugar, and sodium while increasing the dietary fiber and protein content. All meals were free from added preservatives, additives, and sweeteners, and olive oil was the first option for added fat. Considering the increasing recognition of school-based food aid programs as an effective healthy nutrition promotion tool, the results of the present work are of particular importance for public health policies; providing data for national and EU/international food industries regarding the design of healthy meals, particularly those delivered to children/adolescents.

### 4.2. Macronutrients Profile in DIATROFI Meals

#### 4.2.1. FAME Profile

Extra virgin/virgin olive oil has been widely recognized for its well-documented health benefits on the grounds of its high MUFA content along with a considerable amount of phytochemicals with anti-inflammatory and antioxidant properties [24,25]. Therefore, exclusive use of extra virgin/virgin olive oil in all meals distributed to students is a fundamental requirement of the DIATROFI Program, with the addition of other edible oils/fats prohibited due to their evidence-based health implications. On the contrary, food industries almost always use refined oils (i.e., palm oil, cottonseed oil, sunflower oil) as added fat(s), as indicated on the food labels of commercial meals selected for this study. These refined oils are added for sensory-based and palatability reasons, justifying their high SFA, low MUFA content, and probably detectable levels of partially hydrogenated (trans) fatty acids [26]. These fatty acids aggravate children’s health, raising the risk for chronic diseases such as obesity, CVD, diabetes mellitus, and various types of cancer [27,28]. Additionally, glycerol-based process contaminants in palm oil, other common refined vegetable oils, and margarine raise potential health concerns for all young age groups due to their high amounts of 3- and 2-monochloropropanediol (MCPD) and their fatty acids esters [28,29,30]. On the contrary, meals designed for the DIATROFI Program presented a rather different FAME profile (i.e., high MUFA/low SFA content, non-detectable trans fatty acids). Moreover, the inclusion of olive oil, as the only source of added fat, contributes towards ensuring adequate consumption of healthy fat/oils and reaching half the amount of the children-specific recommendations by offering 1–2 servings of olive oil in each meal (children aged 4–8 and 9–13 are advised to consume 2–3 and 3–4 servings of healthy fat daily, respectively) [13].

#### 4.2.2. Added Sugars

Fructose and added sugars, such as sucrose and high fructose corn syrup, are usually mentioned as ingredients in packed/pre-packed products aiming to produce meals attractive for consumers, especially children; however, a predominant health effect of high added sugar consumption in childhood is tooth decay. Most importantly, there is sufficient evidence that decreasing the consumption of added sugars may significantly reduce obesity and obesity-related disease (e.g., metabolic syndrome) prevalence. Sugars are associated with obesity and obesity-related diseases because of their “empty calories”, along with the potential involvement of fructose in metabolic and inflammatory pathways [31,32,33]. Therefore, in all DIATROFI meals, added sugars are limited, as verified through the laboratory analyses, and are solely present in more natural forms, i.e., brown sugar and honey (Table 1). In the case of savory pies and pizzas, the sugar content of DIATROFI meals seems similar or higher compared to their commercially available counterparts; nonetheless, DIATROFI laboratory analysis is applied in already baked ready-to-eat pies and bakery products while the values of commercial pies referred to frozen/unbaked products. Thus, the conversion of the starch content of flour into sugars, probably enhanced during heating treatment/baking [34], along with the less humidity in final baked products, could be responsible for this inconsistency with the other DIATROFI meals outcome.

#### 4.2.3. Dietary Fibers

Most DIATROFI meals contain almost twice as high fiber content compared to commercial products. The consumption of food products with high fiber content is strongly encouraged on the grounds of their long-term health effects. More specifically, minimally processed whole grain products have been recognized for their contribution to retaining normal weight and preventing CVD, several types of cancer, diabetes mellitus, and other chronic diseases [35,36,37]. Considering this association with diseases along with the cardiometabolic harms induced by the high consumption of refined grains, the scientific community has argued for whole grain product consumption as an indispensable part of healthy dietary patterns from childhood to adulthood [36]. To achieve this goal in the context of DIATROFI, only whole wheat bread, bakery products, and savory pies have been distributed to the students, providing a healthier alternative compared to the vast majority of the available-in-the-market products. It should also be noted that children receive daily a fruit serving, further ensuring adequate consumption of dietary fiber and achieving one of the main aims of the Program, which is the limitation of food insecurity [4]. Similar programs that distribute daily fruits and/or vegetables have also proved to effectively reduce food insecurity [38]. Ensuring the sufficient consumption of fruits, vegetables, and whole-wheat products, is a core aim of the DIATROFI Program. Through the daily meals, the Program aims to cover one-third to half of the recommended amount of whole grain products daily by providing two servings of whole-grain foods (for 4–8 years and 9–13 years old children, it is recommended to consume 4–5 and 5–6 servings of mostly whole grain products, respectively) [13]. Furthermore, the Program also offers daily 1–2 servings of fruit (depending on the size) and 0.5–1 serving of vegetables incorporated in the meal and assists children in meeting their recommended daily intake of fruits and vegetables (for 4–8 years and 9–13 years old children it is recommended to consume 1–2 and 2–3 servings of fruits and 1–2 and 2–3 servings of vegetables, respectively) [13].

### 4.3. Micronutrients in DIATROFI Meals

#### Sodium/Salt

Among the strict requirements of DIATROFI meals is their low salt/sodium content, which is in stark contrast with commercial meals, which use it in large quantities, usually for marketing reasons. Our study found that, overall, DIATROFI meals contain less sodium. High salt intake in childhood/adulthood predisposes an individual to various long-term aggravating health effects, including elevated blood pressure, CVD, several types of cancer, and respiratory illnesses [39]. Moreover, eating patterns present later in the life course are determined by the dietary habits adhered to earlier in the life course. Considering that salty taste is a steadily learned preference, the recommendation regarding the limitation of salt intake in adulthood would be more easily met should this habit start earlier in life [40]. Unfortunately, however, literature largely agrees that at preadolescence, children tend to increase their junk food consumption, leading to higher amounts of sodium consumed that could have a detrimental impact on their general eating habits long-term [41]. Current daily salt consumption in most European countries is estimated to be, on average, twice as high as the recommended daily consumption for children and adults, with processed foods being the major source [42]. Among the 2020–2025 global targets is “a 30% relative reduction in mean population intake of salt/sodium” [43]. The current national action plans implemented in Europe underline the significant role of food industries, suggesting the reformulation of packed/pre-packed meals towards less salt/sodium content [44,45]. Consequently, bread, bakery products, and savory pies produced in DIATROFI represent an example of good industrial practice towards the production of packed/pre-packed meals; the DIATROFI whole grain bread, in particular, is an example of good practice as it contained almost one-third of the sodium in other whole bread products.

### 4.4. List of Ingredients for Meal Preparation

#### 4.4.1. Processed Meat/Meat Products & Processed Cheese

An important difference between ready-to-eat meals delivered in the context of DIATROFI and commercial products is the non-inclusion of processed meat products. An evidence-based association of processed meat with premature death, CVD, and mortality has been demonstrated [46]. Furthermore, in 2015 the International Agency for Research on Cancer working group classified processed meat as Group 1, “carcinogenic to humans” [47]. The pathophysiological pathways through which this meat subtype exerts its aggravating role seem to start from its high content of sodium and preservatives (i.e., N-nitroso compounds) [46]. Considering its strong contribution to morbidity and mortality, recommendations note avoiding processed meat consumption early in the life course. Additionally, no processed or melted cheeses are included in DIATROFI meals. This is due to the preparation steps used for their production (i.e., mixing, emulsifying, and blending several types of cheeses via a thermal process) are almost always accompanied by the addition of cream/butter, coloring agents, and emulsifiers leading to a nutritionally suboptimal product [48].

#### 4.4.2. Preservatives and Other Additives

In DIATROFI meals, in contrast with the vast majority of commercially available products, no preservative additions are permitted, based on the literature for their potential health risks. For instance, sorbic/benzoic acids and their respective salts, despite their recognition as “safe for consumption for general population within specific limits”, have been associated with several health implications, including hypersensitivity/allergenicity effects [30,49] and increased hyperactivity for children [50,51]. Similar health outcomes are reported for other “safe for public consumption” food additives and preservatives [50]. Additionally, among the additives widely used in commercial products for their sensory-based and meal structure properties, glutamic acid, glucuronic acid, and all artificial colors possess plausible health implications [52,53]. Monosodium glutamate has been linked with headaches, asthma, and learning difficulties [52,54], while artificial food colors have been linked with adverse behavioral changes in children with and without attention-deficit/hyperactivity disorder [53]. Thus, their addition to all DIATROFI meals is also forbidden.

#### 4.4.3. Sweeteners

As per protocol, all DIATROFI meals are free of all sweeteners, regardless of their classification as natural or artificial. The WHO advises against the use of sugar alternatives in commercially available products or for home use in foods for weight control [55]. According to the WHO’s recent systematic review, examining the effect of synthetic and naturally occurring or modified non-nutritive sweeteners on controlling body weight, sweeteners consumption does not provide any long-term benefit in reducing body fat in adults or children and does not reduce the risk of non-communicable diseases [56]. More specifically, there is no substantial long-term health benefit in children [56]. Nowadays, food manufacturers replace sugar with artificial or natural sweeteners aiming for a seemingly healthier alternative product, which consumers may perceive as an option with added health benefits or with natural ingredients in the case of natural sweeteners [57].

### 4.5. DIATROFI Meal Adaptations: Success and Challenges

Throughout the DIATROFI program’s existence, enormous efforts have been made by the research team in order to improve the quality of the meals despite the evolving challenges. Efforts were concentrated on decreasing the total fat, SFA, sugar, and salt content of DIATROFI meals while increasing the protein and fiber content; in other words, the quality of the food was considered in its entirety rather than focusing on a single nutrient. Furthermore, it is challenging to monitor the effects of these adaptations to the DIATROFI meals on children’s health due to the compounding factors that may confound the effects. The DIATROFI Program’s experience with food reformulation aligns with that of the literature, as it is a gradual process with small improvements, with a bigger impact on population health when the majority of food products are reformulated [58]. Promoting the food reformulation of most food products ensures that the entire population can benefit in the long term; however, in practice, there is variability in the nutrient composition of similar food products [59]. Even more striking is that it is increasingly common that only a subset of food products are reformulated, normally expensive food products, further deepening disparities regarding population health [58]. This gives individuals only with high incomes the opportunity to choose to eat healthy regularly and benefit from reformulated food projects. This is especially relevant to the DIATROFI project, as this project is targeted toward schools of low socioeconomic status throughout Greece, where families have an increased risk of experiencing food insecurity and whose access to healthy, affordable foods is generally low [60]. Food insecurity has steadily increased in recent years in Greece, rising to sixth place in 2019 in Europe [61]. The rising costs of healthy eating habits, in combination with inflation post-COVID, has not only contributed to the food insecurity experienced by families and their ability to purchase quality food but has also impacted the manufacturers’ ability to produce the same high-quality food for the children at low costs. The DIATROFI research team has put tremendous effort into adapting the food delivered daily to the children in order to maintain the high-quality nutrient profile desired while attempting to maintain low costs for the project.

### 4.6. Limitations

Some limitations should be addressed for a more conscious interpretation of the outcomes. Regarding the laboratory analysis, the intra-laboratory reproducibility of the outcomes could not be adequately evaluated, as only three measures of each food item were performed (Food Laboratory and analytical methods) [20]. Moreover, in 2023 the DIATROFI team did not perform any laboratory nutrient analysis on the food items delivered. Regarding the sodium in DIATROFI meals, the exact content from food databases could not be calculated, as the manufacturer only provided a range for its content (e.g., <0.1 g). For the rest of the nutrients, a similar procedure was performed in the previous school years of the Program with a high level of agreement with the current results. The whole FAME profile was not available for the commercial products as this is not mandatory (according to the EU Regulation 1169/2011 [8]) information for food labels; hence, solely SFA content is required to be illustrated in the nutrition facts, was accessible for comparison with the respective DIATROFI meals. Only frozen vegetable pies and pizzas were available in the market with clear food labeling; thus, the nutrients illustrated on the food label are to reach higher levels in the baked product.

## 5. Conclusions

The principal findings of the present work suggest an example of good practice in the field of school feeding programs regarding the design and production of meals according to the principles of healthy nutrition. Most importantly, DIATROFI meals present indicative discrepancies with similar commercial products. In a world with extremely high availability and marketing of unhealthy food choices, especially to children and adolescents, food industries should reformulate their products considering the role that early eating habits play in long-term health. Therefore, it is the national policy-makers’ responsibility of each country to establish specific standards that will ensure the availability of healthier food choices in the market and school settings as a part of a general action plan regarding the amelioration of health status in the entire population.

## Figures and Tables

**Table 1:** Nutrient content of DIATROFI traditional pies based on three different evaluation methods; a. laboratory analysis, b. national and international databases, and c. food labeling.

Meal	Total Fat (g/100 g)	SFA (g/100 g)	Carbohydrates (g/100 g)	Total Sugars (g/100 g)	Protein (g/100 g)	Dietary Fiber (g/100 g)	Sodium (mg/100 g)
**Spinach pie**							
2016 Laboratory Analysis	9.9	0.9	31.0	1.5	5.7	3.8	388
2016 Food databases	10.3	1.6	32.1	1.0	6.4	3.8	- ^†^
2016 Nutrition label	11.1	1.5	28.8	1.3	3.9	2.3	400
2016 Similar commercial products (*n* = 5)	13.5	4.0	25.4	1.3	4.5	1.5	395
2023 Food databases	6.3	0.9	23.1	0.6	6.9	6.2	- ^†^
2023 Nutrition label	6.5	0.9	21.7	0.7	4.4	4.4	360
2023 Similar commercial products (*n* = 4)	17.1	2.9	26.5	2.6	6.5	2.5	343
**Spinach pie with feta cheese**							
2016 Laboratory Analysis	11.3	1.9	28.2	2.7	7.9	3.2	364
2016 Food databases	12.5	3.0	31.7	1.5	8.1	3.7	- ^†^
2016 Nutrition label	12.0	2.5	28.0	1.2	5.2	2.1	430
2016 Similar commercial products (*n* = 4)	18.7	5.1	25.6	1.3	5.9	1.5	415
2023 Food databases	10.0	1.1	22.8	0.7	7.8	5,3	- ^†^
2023 Nutrition label	9.7	0.9	21.1	0.6	6.9	3.8	360
2023 Similar commercial products (*n* = 5)	16.7	4.1	28.8	1.9	5.8	1.6	388
**Leek pie with feta cheese**							
2016 Laboratory Analysis	10.1	1.9	29.5	3.2	8.5	3.2	360
2016 Food databases	12.5	3.0	33.1	1.7	7.6	3.6	- ^†^
2016 Nutrition label	11.1	2.9	29.1	0.8	5.6	2.0	400
2016 Similar commercial products (*n* = 6)	14.8	5.4	24.2	1.4	7.2	1.5	459
2023 Food databases	7.7	1.1	23.7	1	5.0	6.7	- ^†^
2023 Nutrition label	7.1	1	22.4	1	4.1	5	320
2023 Similar commercial products (*n* = 4)	15.4	4.3	28.4	1.6	7.1	1.8	379
**Pumpkin pie**							
2016 Laboratory Analysis	7.2	2.0	31.6	15.2	5.2	1.9	160
2016 Food databases	7.2	1.7	32.4	15.0	5.2	3.0	- ^†^
2016 Nutrition label	7.3	1.6	30.1	14.4	5.1	2.4	141
2016 Similar commercial products (*n* = 0)	-	-	-	-	-	-	-
Zucchini and feta cheese pie *							
2023 Food databases	9.6	1.8	21.1	0.8	6.3	2.3	- ^†^
2023 Nutrition label	9.5	1.5	21.5	0.77	6.3	2.9	320
2023 Similar commercial products (*n* = 3)	11.4	3.2	28	4.5	6.3	2.1	197
Chicken pie *							
2023 Food databases	13.8	5.1	29.2	3.3	12.2	2.9	- ^†^
2023 Nutrition label	13.5	4.9	28.8	3.4	10.7	2.4	400
2023 Similar commercial products (*n* = 4)	11.9	3.5	27.5	3.1	9.3	2	519

Laboratory analysis assessment method (only 2016): Nutrients are presented as the mean value of the results from the *n* = 3 repeated analyses applied in the same meal kind (2016). Food database: Nutrients are calculated through recipe simulation based on national and international databases. Nutrition label: Nutrient values are selected from the DIATROFI meal nutrition label designed by the food suppliers of the Program. Regarding commercial products, mean values referred to the nutrient content of the selected-by-market ready-to-eat meals based on their nutrition label. ^†^ Sodium/salt content of several DIATROFI meals could not be calculated based on food databases due to inadequate information by food suppliers regarding the exact salt addition to the final product (food suppliers provided a statement as regards the salt/sodium content of the final meal within the accepted-by-Institute limits)—compliance with this technical requirement is verified through unannounced laboratory analyses. * The product was introduced after schoolyear 2016–2017. Abbreviations: Saturated fatty acids (SFA).

**Table 2:** Nutrient content of DIATROFI bakery products based on three different evaluation methods; a. laboratory analysis, b. national and international databases, and c. food labeling.

Meal	Total Fat (g/100 g)	SFA (g/100 g)	Carbohydrates (g/100 g)	Total Sugars (g/100 g)	Protein (g/100 g)	Dietary Fiber (g/100 g)	Sodium (mg/100 g)
**Must cookie**							
2016 Laboratory Analysis	14.8	1.3	59.1	17.3	7.6	4.0	80
2016 Food databases	14.1	1.0	59.3	16.9	7.1	4.2	- ^†^
2016 Nutrition label	15.0	2.7	59.5	18.3	6.9	3.9	91
2016 Similar commercial products (*n* = 3)	17.7	4.4	58.3	21.0	6.6	2.2	84
2023 Food databases	9.6	0.8	44.9	9.5	7.7	4.3	- ^†^
2023 Nutrition label	10.2	1.5	45	9.9	7.6	4	40
2023 Similar commercial products (*n* = 4)	19.8	4.1	63.3	18	6.5	2.7	137
**Raisin bread**							
2016 Laboratory Analysis	2.9	0.4	48.4	11.5	10.3	4.8	220
2016 Food databases	2.7	0.6	57.3	15.1	9.1	6.1	- ^†^
2016 Nutrition label	2.9	0.9	54.2	14.9	9.3	5.0	394
2016 Similar commercial products (*n* = 5)	6.5	1.8	52.9	16.5	8.2	3.1	314
2023 Food databases	2.7	0.6	57.3	15.1	9.1	6.1	- ^†^
2023 Nutrition label	2.9	0.9	54.2	14.9	9.3	5	156
2023 Similar commercial products (*n* = 4)	10.9	3.2	52.7	14.3	10.3	3.3	238
**Cake with vegetables (carrot)**							
2016 Laboratory Analysis	16.8	2.5	37.5	10.9	6.5	2.9	276
2016 Food databases	17.2	2.9	39.2	15.4	6.7	3.1	- ^†^
2016 Nutrition label	15.1	3.5	39.8	16.3	6.8	2.7	298
2016 Similar commercial products (*n* = 3) ^	19.8	5.1	50.0	35.0	4.2	2.0	312
2022 Food databases ^‡^	17.2	2.9	39.2	15.4	6.7	3.1	- ^†^
2022 Nutrition label ^‡^	15.1	3.5	39.8	16.3	6.8	2.7	298
2023 Similar commercial products (*n* = 3) ^	14.3	6.7	61.5	36.2	5.2	2	276
**Cake with vegetables (Carrot and beetroot)**							
2016 Laboratory Analysis	14.1	1.6	43.3	13.2	6.7	3.3	333
2016 Food databases	17.8	2.5	42.5	16.1	6.8	3.1	- ^†^
2016 Nutrition label	16.7	3.4	37.3	15.2	6.9	3.1	272
2016 Similar commercial products (*n* = 3) ^	19.8	5.1	50.0	35.0	4.2	2.0	312
2022 Food databases ^‡^	17.8	2.5	42.5	16.1	6.8	3.1	- ^†^
2022 Nutrition label ^‡^	16.7	3.4	37.3	15.2	6.9	3.1	272
2023 Similar commercial products (*n* = 3) ^	14.3	6.7	61.5	36.2	5.2	2	276

Laboratory analysis assessment method (only 2016): Nutrients are presented as the mean value of the results from the *n* = 3 repeated analyses applied in the same meal kind. Food database: Nutrients are calculated through recipe simulation based on national and international databases. Nutrition label: Nutrient values are selected from the DIATROFI meal nutrition label designed by the food suppliers of the Program. Regarding commercial products, mean values referred to the nutrient content of the selected-by-market ready-to-eat meals based on their nutrition label. ^†^ Sodium/salt content of several DIATROFI meals could not be calculated based on food databases due to inadequate information by food suppliers regarding the exact salt addition to the final product (food suppliers provided a statement as regards the salt/sodium content of the final meal within the accepted-by-Institute limits)—compliance with this technical requirement is verified through unannounced laboratory analyses. ^‡^ These products were provided daily across all schools at schoolyear 2021–2022 but were provided to only a limited extent in 2022–2023. ^ Due to the limited availability of similar products in the market and similar recipe profile, the same products were used as the comparison groups. Abbreviations: Saturated fatty acids (SFA).

**Table 3:** Nutrient content of DIATROFI whole wheat bread and dough products based on three different evaluation methods; a. laboratory analysis, b. national and international databases, and c. food labeling.

Meal	Total Fat (g/100 g)	SFA (g/100 g)	Carbohydrates (g/100 g)	Total Sugars (g/100 g)	Protein (g/100 g)	Dietary Fiber (g/100 g)	Sodium (mg/100 g)
**Whole grain bread**							
2016 Laboratory Analysis	3.9	0.6	48.0	2.0	10.0	4.6	430
2016 Food databases	3.4	0.5	52.8	1.5	9.5	5.4	501
2016 Nutrition label	2.7	0.3	48.4	2.4	10.0	4.6	500
2016 Similar commercial products (*n* = 5)	5.3	1.6	40.9	5.2	10.9	6.1	562
2023 Food databases	1.2	0.3	43.8	0.7	9.5	5.3	200
2023 Nutrition label	1	0.3	45.7	0.5	9.1	6	160
2023 Similar commercial products (*n* = 5)	4.1	0.7	42.1	4.5	10.1	5	541
Whole grain pizza slice with vegetables *							
2023 Food databases	10.8	4	24.5	3.1	8.6	2.0	- ^†^
2023 Nutrition label	10	3.9	24	2.8	8.5	1.9	328
2023 Similar commercial products (*n* = 4)	8.5	3.2	28.5	2.9	6.2	1.9	490
Whole grain pizza slice with baked chicken *							
2023 Food databases	10.2	4.1	22.4	2.7	13.4	2.4	- ^†^
2023 Nutrition label	9.8	3.9	22	2.4	13	2.3	284
2023 Similar commercial products (*n* = 4)	8.3	4	28.1	3.9	11.8	0.5	595

Laboratory analysis assessment method (only 2016): Nutrients are presented as the mean value of the results from the *n* = 3 repeated analyses applied in the same meal kind. Food database: Nutrients are calculated through recipe simulation based on national and international databases. Nutrition label: Nutrient values are selected from the DIATROFI meal nutrition label designed by the food suppliers of the Program. Regarding commercial products, mean values referred to the nutrient content of the selected-by-market ready-to-eat meals based on their nutrition label. ^†^ Sodium/salt content of several DIATROFI meals could not be calculated based on food databases due to inadequate information by food suppliers regarding the exact salt addition to the final product (food suppliers provided a statement as regards the salt/sodium content of the final meal within the accepted-by-Institute limits)—compliance with this technical requirement is verified through unannounced laboratory analyses. * The product was introduced after schoolyear 2016–2017. Abbreviations: Saturated fatty acids (SFA).

**Table 4:** Nutrient content of DIATROFI sandwiches based on three different evaluation methods; a. laboratory analysis, b. national and international databases, and c. food labeling.

Meal	Total Fat (g/100 g)	SFA (g/100 g)	Carbohydrates (g/100 g)	Total Sugars (g/100 g)	Protein (g/100 g)	Dietary Fiber (g/100 g)	Sodium (mg/100 g)
**Chicken and tomato sandwich ^**							
2016 Laboratory Analysis	2.4	0.6	28.6	3.3	12.3	2.4	388
2016 Food databases	3.0	0.6	27.2	2.7	13.5	2.4	- ^†^
2016 Nutrition label	2.4	0.5	33.1	2.6	14.9	2.9	315
2016 Similar commercial products (*n* = 6)	8.4	2.4	24.2	2.2	12.5	1.7	459
**Cheese and cucumber or tomato sandwich ^**							
2016 Laboratory Analysis	10.5	6.8 ^†^	30.3	2.1	10.3	3.5	479
2016 Food databases	10.9	5.8	32.9	1.8	10.2	3.5	- ^†^
2016 Nutrition label	10.5	6.8	30.3	2.1	10.3	3.5	479
2016 Similar commercial products (*n* = 4)	9.6	4.2 ^†^	24.0	2.8	12.6	1.6	570
**Whole wheat bread with roasted vegetables and cheese ^**							
2016 Laboratory Analysis	10.2	4.9	19.8	3.7	18.5	5.7	352
2016 Food databases	9.8	3.9	28.9	3.5	11.5	4.7	- ^†^
2016 Nutrition label	10.1	3.8	26.3	1.9	10.1	3.4	656
2016 Similar commercial products (*n* = 0)	-	-	-	-	-	-	-
**Egg and olive-oil-based sauce sandwich ^**							
2016 Laboratory Analysis	8.9	1.9	32.6	1.6	10.6	4.6	300
2016 Food databases	9.1	1.8	33.0	1.9	11.1	4.7	- ^†^
2016 Nutrition label	9.0	2.8	31.0	2.7	11.7	3.2	717
2016 Similar commercial products (*n* = 5)	10.1	2.0	21.9	1.8	10.3	1.9	313

Laboratory analysis assessment method: Nutrients are presented as the mean value of the results from the *n* = 3 repeated analyses applied in the same meal kind. Food database: Nutrients are calculated through recipe simulation based on national and international databases. Nutrition label: Nutrient values are selected from the DIATROFI meal nutrition label designed by the food suppliers of the Program. Regarding commercial products, mean values referred to the nutrient content of the selected-by-market ready-to-eat meals based on their nutrition label. ^†^ Sodium/salt content of several DIATROFI meals could not be calculated based on food databases due to inadequate information by food suppliers regarding the exact salt addition to the final product (food suppliers provided a statement as regards the salt/sodium content of the final meal within the accepted-by-Institute limits)—compliance with this technical requirement is verified through unannounced laboratory analyses. ^ These products were either discontinued due to budgetary limitations or were provided to a limited extent at schoolyears 2021–2022 and 2022–2023. Abbreviations: Saturated fatty acids (SFA).

## Data Availability

The data presented in this study are available on request from the corresponding author.
